# Multi‐Omics Mendelian Randomization Prioritizes Candidate Druggable Genes for Major Depressive Disorder

**DOI:** 10.1111/gbb.70079

**Published:** 2026-11-09

**Authors:** Huijing Zhou, Qiang Liu, Hao Yan, Guixiang Jin, Wei Zhou, Xiaomin Yin, Nai Wang, Minhui Zheng, Haihong Zheng, Yiming Wu

**Affiliations:** ^1^ Department of Psychiatry Mental Health Center Affiliated to Shanghai University of Medicine & Health Sciences (Shanghai Yangpu Mental Health Center) Shanghai China

**Keywords:** colocalization, druggable genes, eQTL, major depressive disorder, Mendelian randomization, pQTL, psychiatric genetics

## Abstract

This study aimed to prioritize candidate druggable genes for major depressive disorder (MDD) using Mendelian randomization (MR) integrated with expression (eQTL) and protein quantitative trait loci (pQTL) data. We conducted a systematic MR analysis combining *cis*‐eQTL and *cis*‐pQTL data from blood and brain tissues to assess the causal effects of druggable genes on MDD, using FinnGen R9 GWAS summary statistics. Bayesian colocalization analysis confirmed shared genetic variants, while sensitivity analyses (MR‐Egger, weighted median, MR‐PRESSO) tested robustness. A phenome‐wide MR (Phe‐MR) assessed broader trait associations. To evaluate translational potential, we mapped candidate genes to known drugs using DrugBank, ChEMBL, and ClinicalTrials.gov.Six candidate druggable genes (MAPK3, PSMB9, SIGLEC9, MST1, BTN3A1, and TAPBP) showed significant associations with MDD across multiple eQTL and pQTL datasets. These genes are involved in biological processes related to neuroinflammation, immune regulation, and apoptosis. Colocalization analyses supported shared genetic signals between the identified genes and MDD, while phenome‐wide analyses identified associations with additional diseases and traits that may be relevant for future translational research. Through MR analysis, six candidate druggable genes were prioritized from 6889 potential druggable genes as significantly associated with MDD. Among them, MAPK3, MST1, and SIGLEC9 showed consistent evidence across multiple analyses and emerged as genetically supported candidate targets for future investigation.

## Graphical abstract

A multi‐omics Mendelian randomization framework integrating blood and brain eQTL/pQTL data with FinnGen GWAS identified six druggable genes causally associated with major depressive disorder. Colocalization and phenome‐wide analyses supported shared genetic architecture and safety profiles, highlighting immune‐inflammatory pathways as promising therapeutic targets.

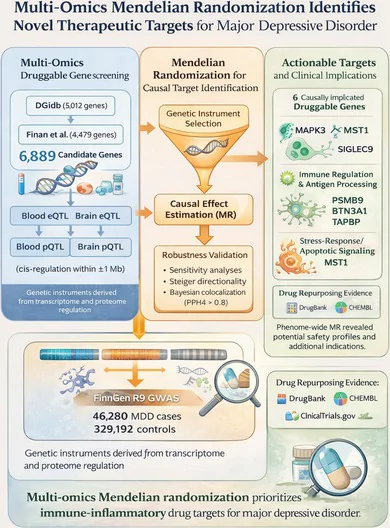

## 1 Introduction

Major depressive disorder (MDD), as defined by the Diagnostic and Statistical Manual of Mental Disorders, Fifth Edition (DSM‐5) [1], is characterized by persistent depressed mood, diminished interest in activities, and recurrent thoughts of death. This debilitating condition affects approximately 185 million people globally [2] and has been a significant public health challenge over the past three decades, profoundly impacting quality of life and healthcare systems worldwide [3]. In 2019, depressive disorders ranked as the 13th leading cause of DALYs (disability‐adjusted life years). This metric combines years of life lost due to premature mortality and years lived with disability. Depressive disorders also showed higher prevalence and disability weights than many other diseases [4]. Despite available treatments, more than one‐third of patients fail to achieve adequate responses to multiple first‐line therapies. Evidence‐based guidelines, such as those by the Canadian Network for Mood and Anxiety Treatments (CANMAT), emphasize the urgent need for novel and more effective treatments [5]. MDD is increasingly recognized as involving both neurobiological and systemic components, including neuroinflammation, immune dysregulation, and apoptosis [6]. These processes present potential entry points for novel therapies targeting druggable genes. By integrating multi‐omics data, such as expression quantitative trait loci (eQTL) and protein quantitative trait loci (pQTL), with Mendelian randomization (MR) analysis, researchers can uncover causal genetic drivers of MDD and their therapeutic relevance. The use of tissue‐specific *cis*‐eQTL and *cis*‐pQTL data derived from blood and brain samples facilitates the identification of biologically meaningful targets [7].

The rapid advancement of genome‐wide association studies (GWASs) has created unprecedented opportunities for drug development in complex diseases. When combined with MR and multi‐omics datasets, these approaches provide innovative methods for identifying therapeutic genes and pathways. This framework not only prioritizes drug targets for conditions like cardiovascular and metabolic disorders but also demonstrates broad applicability in psychiatry [8]. Genetically supported target genes significantly enhance the success rate of drug development [9]. To address the gaps in translating genetic discoveries into clinical applications, this study adopts a systematic MR framework to identify druggable target genes for MDD. Leveraging insights from genomic, transcriptomic, and proteomic datasets, it explores the causal relationships between candidate genes and MDD. Bayesian colocalization analysis confirms their genetic foundations, while phenome‐wide MR (Phe‐MR) analysis evaluates their broader phenotypic effects. The ultimate goal is to prioritize candidate therapeutic targets for MDD and facilitate future translational research based on genetic evidence.

## 2 Material and Methods

The conceptual framework of this study is illustrated in Figure 1.

**FIGURE 1:**
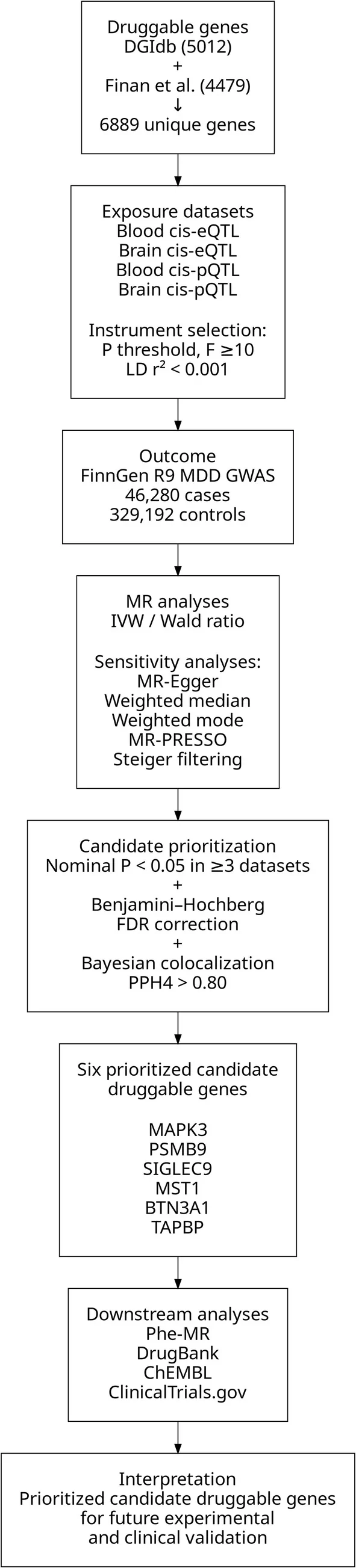
Initially, we obtained 6889 known druggable genes by integrating data from the DGIdb database and the study by Finan et al. We utilized *cis*‐eQTL and *cis*‐pQTL data from human blood and brain tissues to construct an analysis framework for druggable genes. This involved filtering independent genetic variants significantly associated with druggable gene expression (as instrumental variables, IVs) located within a 1 Mb range upstream and downstream of the coding sequence. The filtering criteria included genome‐wide significance thresholds (*p* < 5 × 10^−8^ for blood *cis*‐eQTL, *p* < 5 × 10^−5^ for brain *cis*‐eQTL and blood/brain *cis*‐pQTL), *F*‐statistics ≥ 10, and linkage disequilibrium (LD) thresholds (*r* ^2^ < 0.001). In the Mendelian randomization (MR) analysis, we identified six druggable genes (MAPK3, BTN3A1, PSMB9, TAPBP, SIGLEC9, MST1) with significant causal associations with depression (*p* < 0.05) across at least three exposures. Bayesian colocalization detected shared causal variants. Phe‐MR analysis using UK Biobank GWAS data (767 phenotypes) evaluated potential side effects or additional therapeutic indications of these genes. Drug prediction used relevant databases, identifying 12 drugs targeting 5 of the identified genes, providing insights into their therapeutic potential and clinical development prospects.

### 2.1 Ethics Statement

No additional ethics review was required for this study, as it exclusively analyzed aggregated genetic data in accordance with the Declaration of Helsinki.

### 2.2 Data Sources

#### 2.2.1 Druggable Gene Identification

In this study, druggable genes were sourced from the Drug‐Gene [10] Interaction Database (DGIdb v5.0.2, https://www.dgidb.org/downloads) and the research conducted by Finan C. et al. [11] (Table 1; S1 and S2). DGIdb is an open‐access resource designed to aggregate and integrate comprehensive data related to genes, drugs, and their interactions. DGIdb 5.0.2 collects data from various sources such as ChemIDplus, Drugs@FDA, HemOnc, NCIt, and RxNorm, and enhances the grouping and annotation of drugs and genes, particularly in terms of regulatory approval statuses for therapeutic drugs. The latest version also incorporates a GraphQL API, enabling more flexible and efficient data queries tailored to diverse research needs [10]. Furthermore, the druggable genes identified by Finan et al. were incorporated into our analysis due to their connection between GWAS‐identified loci associated with complex diseases and druggable genes, thus aiding in the discovery and validation of potential drug targets [11]. DGIdb contains 5012 druggable genes, while Finan's dataset provides 4479 druggable genes.

**TABLE 1:** Data sources for the MR analysis in the current study.

Type of dataset	Data subtype	Source	Sample size	Population	Download site
Druggable genome	DGIdb 5.0.2	Cannon et al. [10]			https://www.dgidb.org/downloads/
	Prior druggable gene	Finan et al. [11]			Finan C, et al. PMID: 28356508.
QTL datasets	Brain *cis*‐eQTL	Qi et al. [12]	2865	European	https://yanglab.westlake.edu.cn/
	Brain *cis*‐pQTL	Yang et al. [13]	380	European	Yang et al. PMID: 34239129.
	Blood *cis*‐eQTL	eQTLGen Consortium	31,684	European	https://eqtlgen.org/
	Blood *cis*‐pQTL	deCODE	35,559	European	https://www.decode.com/summarydata/
GWAS summary	Depression	Finngen R9	46280case: 329192con	European	https://storage.googleapis.com/finngen‐public‐data‐r9/
	1419 Phenotypes	UK Biobank	408,961	European	https://www.leelabsg.org/resources

*Note:* The study utilized multiple datasets to identify and validate druggable genes associated with depression. The druggable genome data were sourced from DGIdb 5.0.2 (Cannon et al. [10]) and prior research (Finan et al. [11]). The QTL datasets included brain *cis*‐eQTL (Qi et al. [12]; *n* = 2865) and brain *cis*‐pQTL (Yang et al. [13]; *n* = 380), as well as blood *cis*‐eQTL from the eQTLGen Consortium (*n* = 31,684) and blood *cis*‐pQTL from deCODE (*n* = 35,559). GWAS summary statistics for depression were obtained from FinnGen R9 (46,280 cases, 329,192 controls) and UK Biobank (1419 phenotypes; *n* = 408,961). All datasets focused on European ancestry populations. Detailed dataset information, including sources and download sites, is provided in the table.

#### 2.2.2 eQTL and pQTL Datasets

To identify potential drug targets for MDD, we focused on *cis*‐regulatory elements, specifically analyzing genetic variants located within a 1 Mb range on either side of the coding sequences of druggable genes, as these elements exert more direct and specific biological effects compared to trans‐regulatory elements [14]. For this analysis, we utilized *cis*‐eQTL and *cis*‐pQTL data from human blood and brain tissues. The blood *cis*‐eQTL data, which encompasses information on 16,989 gene transcripts from 31,684 individuals, was sourced from the eQTLGen Consortium [15]. Similarly, the brain *cis*‐eQTL data were obtained from BrainMeta [12], and it includes 2865 brain cortex samples from 2443 unrelated individuals of European ancestry, analyzed using RNA sequencing and genome‐wide SNP data. Furthermore, the blood *cis*‐pQTL data were derived from a large‐scale study involving 35,559 Icelanders, providing summary statistics for genetic associations related to 4907 circulating proteins [16]. Finally, the brain *cis*‐pQTL data were sourced from a study led by Dr. Chengran Yang and colleagues at Washington University School of Medicine [13]. This dataset includes 380 brain cortex samples, analyzed to measure the abundance of 1079 proteins using an aptamer‐based platform. The samples were collected from 1537 participants of European ancestry.

#### 2.2.3 GWAS Datasets

GWAS summary statistics for MDD were obtained from the FinnGen R9 dataset, which is part of a large public‐private partnership aimed at understanding the genetic basis of diseases by analyzing genome and health data from 500,000 Finnish biobank donors. The FinnGen R9 dataset includes 46,280 cases and 329,192 controls, focusing on individuals of European ancestry. This project is expanding to explore disease progression and biological mechanisms, providing a world‐class resource for breakthroughs in disease prevention, diagnosis, and treatment.

#### 2.2.4 Instrument Selection

MR uses single nucleotide polymorphisms (SNPs) that are strongly associated with the exposure as instrumental variables (IVs) to assess the causal effect of the exposure on the outcome. Eligible IVs must meet three assumptions [17]: (1) IVs must be significantly associated with the exposure; (2) IVs must be independent of any confounders; (3) IVs must affect the outcome only through the exposure and not via other pathways. First, we cross‐referenced 6889 potential druggable genes with the human blood and brain eQTL/pQTL datasets to obtain eQTL/pQTL data for the druggable genes (Figure 1). Next, we extracted *cis*‐regulatory genetic variants closely associated with druggable gene expression, which were located within 1 Mb on either side of the coding sequences of the druggable genes. To minimize the effects of pleiotropy and ensure compliance with the three MR assumptions, that is, that IVs should influence the outcome only through the exposure and not through other pathways, we applied different genome‐wide significance thresholds: *p* < 5 × 10^−8^ for blood eQTL, and *p* < 5 × 10^−5^ for blood pQTL, brain eQTL, and brain pQTL, while applying an *F*‐statistic ≥ 10 to select strong IVs [18]. Strong instrumental variables (*F*‐statistic ≥ 10) were selected to minimize weak instrument bias and improve the reliability of the MR analyses. Finally, to ensure that each selected significant SNP was independent and to exclude pleiotropic effects caused by linkage disequilibrium (LD), we set the LD coefficient *r* ^2^ to 0.001, with an LD window size of 10 Mb, and used the clumping function in the “TwoSampleMR” package to obtain the IVs [19]. To minimize weak instrument bias, only instrumental variables with an *F*‐statistic ≥ 10 were retained for MR analyses, ensuring adequate instrument strength and improving the reliability of the causal estimates.

### 2.3 MR Analysis and Steiger Filtering Analysis

Upon identifying the instrumental variables, effect estimates for the corresponding variants or suitable proxy variants were extracted from the MDD GWAS dataset, and exposure–outcome associations were harmonized. The causal effects were estimated using the inverse‐variance weighted (IVW) method under a random‐effects model when multiple instrumental variables were available, whereas the Wald ratio method was applied for analyses involving a single instrumental variable. To assess the robustness of the causal estimates, complementary sensitivity analyses were performed using the MR‐Egger, weighted median, and weighted mode methods [20]. Heterogeneity was evaluated using Cochran's *Q* statistic under the IVW model, whereas horizontal pleiotropy was assessed using the MR‐Egger intercept test [21]. The MR‐PRESSO global test was applied to detect potential outlier SNPs, and any significant outliers identified were removed from the analysis [22]. Steiger filtering was further performed to evaluate the direction of causality. For heterogeneity and pleiotropy tests, uncorrected *p* values < 0.05 were considered statistically significant. In addition, to account for multiple comparisons, the primary MR *p* values within each exposure dataset were adjusted using the Benjamini–Hochberg false discovery rate (FDR) procedure, and the FDR‐adjusted *p* values are provided in the Tables (S8–S11). Causal directionality was assessed using the Steiger analysis method [23]. We used Steiger filtering in the “TwoSampleMR” R package to reduce reverse causality risk. The results were classified into three categories to facilitate interpretation: “true” if the effect direction was from exposure to outcome (*p* < 0.05); “false” if the effect was reversed (*p* < 0.05); and “uncertain” if *p* ≥ 0.05. All analyses were performed using R software (version 4.1.2) with relevant R packages (TwoSampleMR, MRPRESSO, and Rmediation).

### 2.4 Bayesian Colocalization

Bayesian colocalization is a statistical method used to determine whether the observed association signals between two traits (Trait 1: potential druggable genes, Trait 2: MDD) stem from the same genetic variant. This is achieved by calculating the posterior probabilities for five different hypotheses [24]: (i) PPH0, no association between the genetic variant and either trait; (ii) PPH1, the variant is associated only with Trait 1; (iii) PPH2, the variant is associated only with Trait 2; (iv) PPH3, both traits are associated with the variant, but through different genetic variants; and (v) PPH4, both traits are associated with the same genetic variant. If the posterior probability PPH4 exceeds 80%, it is considered that the potential druggable genes and MDD share the same genetic variant [24]. We conducted Bayesian colocalization analysis using the “coloc” package in R (http://cran.r‐project.org/web/packages/coloc/).

### 2.5 Candidate Gene Selection Criteria

Candidate genes were prioritized using an integrative strategy rather than a single statistical criterion. First, genes or proteins showing evidence of association with MDD (*p* < 0.05) in at least three independent exposure datasets were selected for further evaluation. To account for multiple comparisons, *p* values obtained from each MR analysis were additionally adjusted using the Benjamini–Hochberg FDR procedure, and the FDR‐adjusted *p* values are provided in Tables S8–S11. The robustness of the identified associations was further evaluated using complementary sensitivity analyses, including MR‐Egger, weighted median, weighted mode, MR‐PRESSO, heterogeneity testing, and Steiger filtering, where applicable. Candidate genes were subsequently prioritized based on the consistency of evidence across multiple independent datasets, complementary sensitivity analyses, and Bayesian colocalization analysis. In addition, all prioritized candidate genes were required to pass Bayesian colocalization analysis with a posterior probability of hypothesis 4 (PPH4) > 0.80, indicating that the genetic association signals for gene expression (or protein abundance) and MDD were likely driven by the same underlying causal variant. This multi‐step prioritization strategy has been recommended for exploratory Mendelian randomization‐based drug target discovery to improve the robustness of candidate selection [25].

### 2.6 Phenome‐Wide Mendelian Randomization (Phe‐MR)

The goal of our Phe‐MR analysis was to investigate the causal relationships between the identified druggable genes and other disease traits, with the aim of evaluating potential associations with other diseases and traits that may be relevant for future translational research and safety assessment. Researchers have utilized the SAIGE (Scalable and Accurate Implementation of Generalized Mixed Models) method to analyze over 1400 binary phenotypes from 408,961 British white participants of European ancestry in the UK Biobank [26]. We obtained 767 non‐depressive disorders or traits with a sample size of more than 500 from the SAIGE GWAS (https://www.leelabsg.org/resource) to ensure statistical robustness (Tables 1 and S14).

### 2.7 Identification of Actionable Drugs for Candidate Genes

To evaluate drug development for the identified druggable genes, we conducted a search in multiple databases, including DrugBank (version 5.1.10, https://go.drugbank.com), ChEMBL (version 33, https://www.ebi.ac.uk/chembl), and ClinicalTrials (https://www.clinicaltrials.gov). This search aimed to identify relevant drug molecule types, therapeutic indications for the drug targets, and ongoing or completed clinical development activities.

## 3 Results

### 3.1 Druggable Genes

We retrieved 5012 potential drug target genes from DGIdb v5.0.2 (Table S1) [10] and identified 4479 druggable genes from the study by Finan et al. (Table S2) [11]. To ensure comprehensive coverage of druggable genes, we combined the two datasets, resulting in a total of 6889 unique genes that were assigned official names by the Human Genome Nomenclature Committee (Table S3).

### 3.2 Candidate Druggable Genes for MDD

We cross‐referenced 6889 potential druggable genes with gene data from human blood and brain tissue eQTL/pQTL datasets, and then extracted genetic variants located within a 1 Mb range on either side of the overlapping druggable gene coding sequences. After IV selection and quality control, we identified 8272 SNPs associated with 3762 druggable genes from blood *cis*‐eQTL, 5321 SNPs related to 3278 druggable genes from brain tissue *cis*‐eQTL, 6512 SNPs linked to 1463 proteins from blood *cis*‐pQTL, and 128 SNPs associated with 123 proteins from brain tissue *cis*‐pQTL. These SNPs served as IVs representing the exposures for MR analysis (Figure 1; Tables S4–S7).

Next, we performed MR analysis on the exposures using the GWAS summary datasets for MDD. We identified 329 and 292 druggable genes from the human blood eQTL and brain eQTL datasets, respectively, as well as 102 and 7 proteins from the human blood pQTL and brain pQTL datasets, respectively, showing a potential causal relationship with MDD (*p* < 0.05, Tables S8–S11).

Ultimately, we identified 6 druggable genes (MAPK3, PSMB9, SIGLEC9, MST1, BTN3A1, TAPBP) that exhibit a causal relationship with MDD (Figure 1). The six candidate genes were prioritized based on the consistency of evidence across multiple independent datasets, sensitivity analyses, and Bayesian colocalization rather than a single statistical criterion.

### 3.3 eQTL Analysis Results

To identify druggable genes associated with MDD, we performed *cis*‐expression quantitative trait loci (*cis*‐eQTL) analysis on both human blood and brain tissues. The significant findings from these analyses are summarized below:

#### 3.3.1 Blood eQTL Analysis

- In the blood eQTL analysis, several genes showed significant associations with MDD, including MAPK3, BTN3A1, PSMB9, TAPBP, and MST1 (Figure 2; Table S8). For example, MAPK3 had a negative effect estimate of −0.053 (95% CI: −0.088 to −0.019) using the inverse variance weighted (IVW) method, while BTN3A1 showed a negative association with an effect estimate of −0.071 (95% CI: −0.115 to −0.026).

**FIGURE 2:**
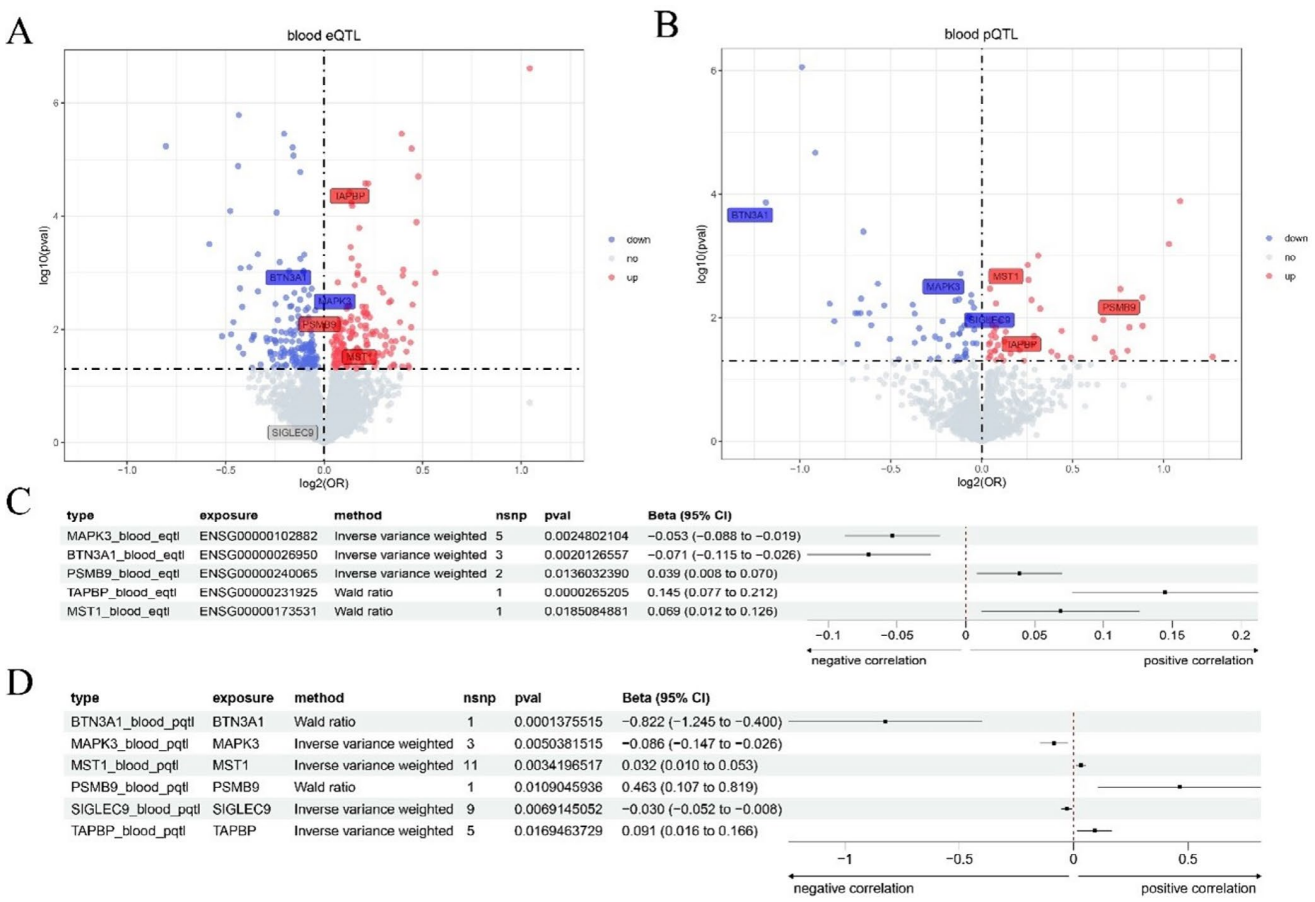
Blood *cis*‐eQTL and *cis*‐pQTL analyses highlight druggable genes linked to MDD. (A) Volcano plot of blood *cis*‐eQTL analysis. The *X*‐axis represents the log2(OR) of gene expression effects on MDD, and the *Y*‐axis represents −log10(*p* value). Genes significantly associated with MDD (*p* < 0.05) are highlighted. TAPBP, PSMB9, and MST1 show positive associations with MDD, while BTN3A1 and MAPK3 display negative associations. Red dots represent upregulated genes, and blue dots indicate downregulated genes. Dashed lines represent thresholds for statistical significance and neutrality in log2(OR). (B) Volcano plot of blood *cis*‐pQTL analysis. MST1, PSMB9, and TAPBP exhibit a positive association with MDD, while BTN3A1, MAPK3, and SIGLEC9 show a significant negative association. (C) Forest plot of *cis*‐eQTL results. MAPK3 (Beta = −0.053, 95% CI: −0.088 to −0.019) and BTN3A1 (Beta = −0.071, 95% CI: −0.115 to −0.026) show negative correlations, while PSMB9 (Beta = 0.039, 95% CI: 0.008 to 0.070), TAPBP (Beta = 0.145, 95% CI: 0.077 to 0.212), and MST1 (Beta = 0.069, 95% CI: 0.012 to 0.126) display positive correlations with MDD. (D) Forest plot of *cis*‐pQTL results. BTN3A1 (Beta = −0.822, 95% CI: −1.245 to −0.400), MAPK3 (Beta = −0.086, 95% CI: −0.147 to −0.026), and SIGLEC9 (Beta = −0.030, 95% CI: −0.052 to −0.008) show a strong negative association with MDD, while MST1 (Beta = 0.032, 95% CI: 0.010 to 0.053), PSMB9 (Beta = 0.463, 95% CI: 0.107 to 0.819), and TAPBP (Beta = 0.091, 95% CI: 0.016 to 0.166) exhibit significant positive correlations.

#### 3.3.2 Brain eQTL Analysis

- In the brain eQTL analysis, similar genes were found to be significantly associated with MDD, such as BTN3A1 and MAPK3 (Figure 3; Table S9). Notably, BTN3A1 showed a strong association, with an effect estimate of −0.073 (95% CI: −0.110 to −0.035), suggesting its role in MDD‐related neural processes.

**FIGURE 3:**
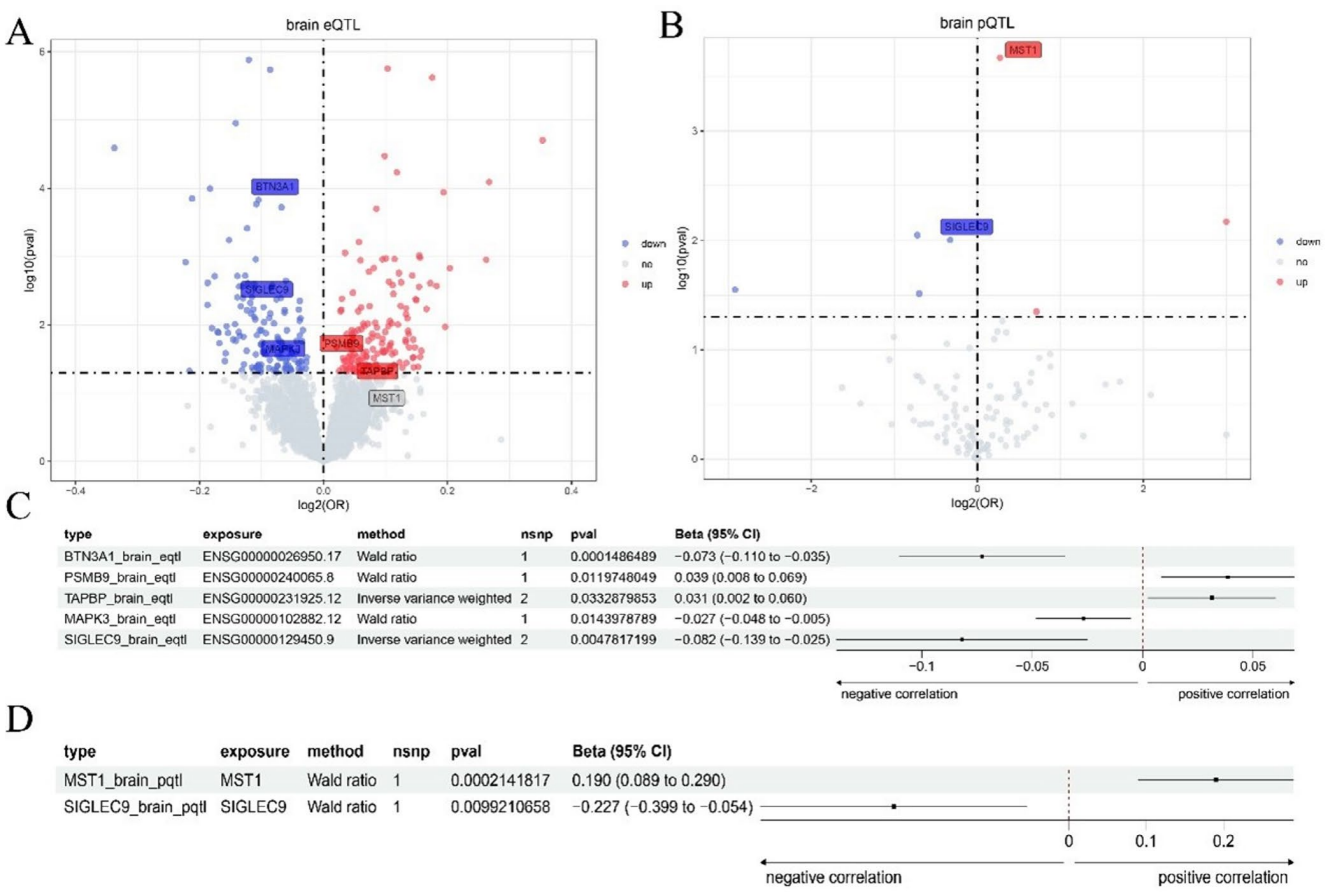
Brain tissue‐based *cis*‐eQTL and *cis*‐pQTL analyses reveal key druggable genes for MDD. (A) Volcano plot of brain *cis*‐eQTL analysis. The *X*‐axis represents the log2(OR) of gene expression effects on MDD, and the *Y*‐axis represents −log10(*p* value). Genes significantly associated with MDD (*p* < 0.05) are highlighted. BTN3A1, SIGLEC9, and MAPK3 show negative associations with MDD, while PSMB9 and TAPBP display positive associations. Red dots represent upregulated genes, and blue dots indicate downregulated genes. Dashed lines represent thresholds for statistical significance and neutrality in log2(OR). (B) Volcano plot of brain *cis*‐pQTL analysis. MST1 exhibits a positive association with MDD, while SIGLEC9 shows a significant negative association. (C) Forest plot of *cis*‐eQTL results. BTN3A1, MAPK3, and SIGLEC9 show negative correlations, while PSMB9 and TAPBP show positive correlations with MDD. (D) Forest plot of *cis*‐pQTL results. MST1 has a positive correlation with MDD (Beta = 0.190, 95% CI: 0.089 to 0.290), and SIGLEC9 shows an opposite trend (Beta = −0.227, 95% CI: −0.399 to −0.054).

### 3.4 pQTL Analysis Results

We also conducted protein quantitative trait loci (*cis*‐pQTL) analysis to examine the relationship between protein abundance of druggable genes and MDD in both blood and brain tissues:

#### 3.4.1 Blood pQTL Analysis

- Key genes such as MAPK3, MST1, and BTN3A1 demonstrated significant associations in the blood pQTL analysis. For example, MAPK3 had a negative effect estimate of −0.086 (95% CI: −0.147 to −0.026), highlighting its potential relevance as a candidate therapeutic target for MDD. (Figure 2, Table S10).

#### 3.4.2 Brain pQTL Analysis

- In the brain pQTL analysis, MST1 and SIGLEC9 were significantly associated with MDD. Notably, SIGLEC9 exhibited an effect estimate of −0.227 (95% CI: −0.399 to −0.054), reinforcing its relevance to the disease mechanism (Figure 3; Table S11).

### 3.5 Volcano Plot Summary

To visualize the differential gene expression and protein abundance, we generated volcano plots for both eQTL and pQTL datasets in blood and brain tissues (Figures 2, 3). The volcano plots provided a graphical representation of significant upregulation or downregulation of the genes:

#### 3.5.1 Blood eQTL

Genes such as MAPK3, PSMB9, and SIGLEC9 were prominently displayed in the volcano plot, indicating significant dysregulation in MDD patients (Figure 2).

#### 3.5.2 Brain eQTL

The volcano plot for brain eQTL highlighted TAPBP (Tapasin binding protein) and SIGLEC9 as showing significant dysregulation (Figure 3).

#### 3.5.3 Blood pQTL

In the blood pQTL analysis, genes like MST1 and BTN3A1 showed significant changes, which were visually depicted in the volcano plot (Figure 2).

#### 3.5.4 Brain pQTL

The volcano plot for brain pQTL depicted significant dysregulation in MST1 and SIGLEC9, further suggesting their role in MDD susceptibility (Figure 3).

### 3.6 Summary of Candidate Druggable Genes

Based on our MR and subsequent analyses, we identified six candidate druggable genes (MAPK3, PSMB9, SIGLEC9, MST1, BTN3A1, TAPBP). These genes demonstrated significance across multiple datasets, and although the effect directions and magnitudes differed between tissues, they were consistently identified in at least three QTL datasets, making them candidate druggable genes for further investigation in MDD.

### 3.7 Colocalization Analysis Results

To further validate whether the association signals of the candidate genes and MDD were derived from the same causal variants, Bayesian colocalization analysis was performed. Strong evidence of colocalization (PPH4 > 0.8) was observed for all six genes across blood and brain eQTL/pQTL datasets (Table S12). For example, BTN3A1 in blood eQTL showed the highest posterior probability (PPH4 = 0.96), TAPBP in brain eQTL reached PPH4 = 0.98, and MST1 in blood eQTL achieved PPH4 = 0.99. Similarly, MAPK3 (PPH4 = 0.86 in blood eQTL; 0.91 in brain eQTL), PSMB9 (PPH4 = 0.88 in blood eQTL; 0.91 in brain eQTL), and SIGLEC9 (PPH4 = 0.89 in brain eQTL; 0.82 in blood pQTL) all demonstrated high posterior probabilities, indicating shared genetic bases with MDD These findings provide additional evidence that the identified association signals may share common genetic variants with MDD and support the prioritization of these genes as candidate druggable genes for further investigation. However, functional studies are required to validate their biological roles and therapeutic relevance.

### 3.8 Phe‐MR Analysis of Candidate Druggable Genes for MDD

To evaluate the potential effects of candidate druggable genes for MDD on other diseases, we conducted a Phe‐MR analysis involving 784 diseases and traits from the UK Biobank. The analysis focused on six candidate genes associated with MDD (Tables S13–S15). The purpose of the Phe‐MR analysis was to determine whether these genes, as drug targets, might lead to adverse side effects or present additional therapeutic opportunities in other diseases.

MAP K3 was found to be negatively associated with rheumatoid arthritis and other inflammatory polyarthropathies (OR = 0.70, 95% CI: 0.59–0.83, *p* < 1E−4), suggesting that drugs targeting MAPK3 might have a protective effect against inflammatory joint diseases. The observed association suggests that MAPK3 may be involved in biological pathways relevant to both MDD and inflammatory diseases. Further studies are required to determine the clinical implications of this finding.

BTN3A1 showed significant associations with multiple diseases. Specifically, BTN3A1 was positively associated with celiac disease (OR = 3.04e−08, *p* < 1E−7) and hematuria (OR = 5.65, *p* < 1E−5), suggesting potential risks related to immune and urinary system diseases if targeted as a drug for MDD. These findings highlight the importance of monitoring potential adverse effects of BTN3A1 in drug development.

PSMB9 was positively associated with Type 1 diabetes (OR = 1.46, *p* < 1E−9) and hyperthyroidism (OR = 1.38, *p* < 1E−5), suggesting that PSMB9 may be involved in biological pathways related to metabolic and endocrine disorders. These findings highlight the importance of considering potential pleiotropic effects when evaluating PSMB9 as a candidate druggable gene. However, the present results do not directly demonstrate therapeutic efficacy or adverse effects, and further experimental and clinical studies are required to clarify the translational implications of targeting PSMB9.

TAPBP showed a negative association with celiac disease (OR = 0.63, *p* < 1E−10), indicating a possible protective role in some immune system diseases. However, TAPBP also showed a positive association with rheumatoid arthritis (OR = 1.21, *p* < 1E−5), suggesting that it might increase the risk of inflammatory conditions. Therefore, the impact of TAPBP on immune‐related diseases should be carefully considered when developing MDD treatments.

MST1 was positively associated with hypertension and angina pectoris (OR = 1.21 and OR = 1.41, *p* < 1E−4, respectively), suggesting an increased risk of cardiovascular diseases when targeted as a drug. Thus, drugs targeting MST1 may require a more thorough safety assessment during the drug development phase.

Overall, the Phe‐MR analysis provided an initial assessment of the potential effects of candidate druggable genes for MDD on other diseases, identifying possible side effects and additional therapeutic effects. These findings are instrumental in balancing the risks and benefits of these genes during drug development, ultimately optimizing treatment strategies for MDD.

### 3.9 Actionable Drugs for Target Genes

We evaluated the pre‐clinical or clinical development activities of the six candidate druggable genes identified for MDD (Table 2). Among these 6 genes, 5 (MAPK3, PSMB9, SIGLEC9, MST1, and BTN3A1) have associated compounds that are either in pre‐clinical or clinical development, while no drugs targeting TAPBP have yet entered clinical trials for depression or related psychiatric conditions.

**TABLE 2:** Identification of actionable drugs for target genes.

Druggable gene	Molecule type	Compounds	Drug group	Action type	Druggable gene molecular function
MAPK3	Small molecule	Sulindac	Approved, investigational	Inhibitor	The protein encoded by this gene is a member of the MAP kinase family. MAP kinases, also known as extracellular signal‐regulated kinases (ERKs), act in a signaling cascade that regulates various cellular processes such as proliferation, differentiation, and cell cycle progression in response to a variety of extracellular signals.
	Small molecule	Arsenic trioxide	Approved, investigational	Inducer	
	Small molecule	Purvalanol	Experimental	Inhibitor	
	Small molecule	Ulixertinib	Investigational	Inhibitor	
	Small molecule	Ravoxertinib	Investigational	Modulator	
PSMB9	Small molecule	Carfilzomib	Approved, investigational	Inhibitor	The proteasome is a multicatalytic proteinase complex with a highly ordered ring‐shaped 20S core structure.
SIGLEC9	Monoclonal antibody	Epratuzumab	Investigational	Antibody; regulator	Predicted to enable monosaccharide binding activity and sialic acid binding activity. Predicted to be involved in cell adhesion. Predicted to act upstream of or within negative regulation of inflammatory response and negative regulation of phagocytosis, engulfment.
	Monoclonal antibody	Inotuzumab ozogamicin	Approved, investigational	Antibody; regulator	
	Monoclonal antibody	Moxetumomab pasudotox	Approved, investigational	Binder; antibody; regulator	
MST1	Small molecule	Fostamatinib	Approved, investigational	Inhibitor	The protein encoded by this gene contains four kringle domains and a serine protease domain, similar to that found in hepatic growth factor.
	Small molecule	Crizotinib	Approved, investigational	Inhibitor	
BTN3A1	Monoclonal antibody	Omburtamab	Investigational	Binder	The butyrophilin (BTN) genes are a group of major histocompatibility complex (MHC)‐associated genes that encode Type I membrane proteins with 2 extracellular immunoglobulin (Ig) domains and an intracellular B30.2 (PRYSPRY) domain.

#### 3.9.1 MAPK3

This gene is targeted by several small molecule inhibitors, including Sulindac and Ulicertinib. These compounds are primarily in the investigational stage, with Sulindac also being approved for other indications. MAPK3, being a member of the MAP kinase family, plays a critical role in regulating cellular proliferation and differentiation, making it a promising target for further exploration in depression treatment.

#### 3.9.2 PSMB9

Carfilzomib, a small molecule inhibitor of PSMB9, is currently approved and in further investigation for other disorders. The proteasome activity regulated by PSMB9 has implications in inflammation and immune response, which are relevant pathways in psychiatric disorders.

#### 3.9.3 SIGLEC9

This gene is targeted by several monoclonal antibodies, including Epratuzumab and Inotuzumab ozogamicin, which are in investigational or approved status for other diseases. SIGLEC9 is involved in cell adhesion and immune regulation, suggesting its potential relevance to immune‐inflammatory pathways implicated in MDD.

#### 3.9.4 MST1

Fostamatinib, a small molecule inhibitor of MST1, is currently approved and undergoing investigation for other diseases. MST1 plays a role in cell signaling pathways associated with neuronal survival, and its modulation could offer therapeutic opportunities for depression.

#### 3.9.5 BTN3A1

Omburtamab, a monoclonal antibody, is in the investigational stage and targets BTN3A1. BTN3A1 is involved in immune regulation, and targeting this pathway might influence neuroinflammatory processes related to MDD.

Phe‐MR associations should not be interpreted as direct evidence of drug efficacy, adverse effects, or clinical outcomes. Instead, these findings provide insight into potential biological relationships and possible pleiotropic effects that may warrant further investigation.

Overall, the Phe‐MR analysis provided an initial assessment of potential associations between the candidate genes and a broad range of diseases and traits. These findings may help identify possible opportunities and challenges for future therapeutic development; however, further validation is required before clinical implications can be inferred.

The identified genes have existing compounds or investigational agents that may facilitate future translational research. However, most of these compounds were developed for non‐psychiatric indications, and their efficacy, safety, blood–brain barrier penetration, and clinical utility in MDD remain largely unknown. Therefore, substantial preclinical and clinical investigation is required before these findings can be translated into therapeutic applications for depression.

## 4 Discussion

MAPK3 (ERK1/2) is a key component of the Ras‐Raf‐MEK‐ERK signaling pathway and plays important roles in cell proliferation, survival, differentiation, synaptic plasticity, and inflammatory responses. In the present study, genetically predicted MAPK3 expression was significantly associated with MDD in both blood and brain datasets, suggesting a potential role in disease pathogenesis. Previous studies have also implicated the ERK/MAPK signaling pathway in several neuropsychiatric disorders, including depression, where altered pathway activity has been associated with neuroinflammation, impaired neuroplasticity, and antidepressant response [27, 28, 29, 30, 31, 32]. Therefore, the present findings are generally consistent with previous evidence supporting the involvement of MAPK signaling in MDD.

Several compounds, including Sulindac, Arsenic trioxide, Purvalanol, Ulixertinib, and Ravoxertinib, have been reported to modulate MAPK3‐related signaling pathways [33, 34]. However, these agents were primarily developed for inflammatory disorders or cancer treatment rather than depression, and their potential utility in MDD remains largely unexplored. In addition, some of these compounds are associated with important safety concerns, including gastrointestinal toxicity, hepatotoxicity, cardiotoxicity, and cytotoxicity, which may limit their potential for psychiatric applications.

Compared with previous studies that mainly reported differential expression or pathway involvement of MAPK signaling in depression, the present study provides additional genetic evidence supporting MAPK3 as a candidate druggable gene through the integration of MR, eQTL, pQTL, and colocalization analyses. Nevertheless, the current findings do not directly demonstrate therapeutic efficacy or confirm MAPK3 as a validated drug target. Further experimental studies and clinical investigations are required to determine whether modulation of MAPK3 can provide therapeutic benefit in patients with MDD.

### 4.1 Role of MST1 in MDD and Its Potential Relevance as a Candidate Therapeutic Target

MST1 (mammalian STE20‐like kinase 1) is a serine/threonine kinase involved in the regulation of apoptosis, oxidative stress responses, inflammation, and cellular homeostasis. In the present study, MST1 showed significant associations with MDD in both blood and brain pQTL datasets, suggesting a potential role in disease pathogenesis. Previous studies have reported that MST1 contributes to neuronal injury, oxidative stress, neuroinflammation, and synaptic dysfunction, all of which have been implicated in the development of depressive disorders [35].

Experimental studies have demonstrated that increased MST1 activity may promote neuronal apoptosis, impair synaptic plasticity, and enhance inflammatory signaling pathways. Conversely, suppression of MST1 has been associated with reduced neuroinflammation and improved neuronal function in preclinical models [36, 37]. These observations are generally consistent with the present genetic findings and support the potential involvement of MST1 in biological processes relevant to MDD.

Several compounds, including Fostamatinib and Crizotinib, have been reported to influence MST1‐related signaling pathways. However, these agents were primarily developed for other clinical indications, including immune‐mediated disorders and cancer, and their potential therapeutic value in depression remains uncertain. Furthermore, both drugs are associated with important adverse effects, including hypertension, thrombocytopenia, gastrointestinal toxicity, visual disturbances, and liver dysfunction, which may limit their applicability in psychiatric disorders.

Compared with previous studies that primarily focused on the role of MST1 in neuronal injury and stress‐related signaling, the present study provides additional genetic evidence supporting MST1 as a candidate druggable gene associated with MDD. Nevertheless, the current findings do not establish MST1 as a validated therapeutic target. Further mechanistic studies, functional experiments, and clinical investigations are required to determine whether modulation of MST1 could provide therapeutic benefit in patients with depression.

### 4.2 Association of SIGLEC9 With Immune Regulation and MDD

SIGLEC9 is an immunoregulatory receptor primarily expressed on monocytes, macrophages, neutrophils, and other myeloid cells, where it contributes to the regulation of inflammatory and immune responses. In the present study, SIGLEC9 showed significant associations with MDD in both eQTL and pQTL analyses across blood and brain tissues, suggesting a potential role in the immune‐related mechanisms underlying depression.

Previous studies have demonstrated that SIGLEC9 participates in the regulation of inflammatory signaling pathways and immune homeostasis [38, 39, 40, 41]. Experimental evidence suggests that soluble Siglec‐9 can suppress inflammatory responses through modulation of NF‐κB signaling, while other studies have reported associations between Siglec‐9 and cognitive function, neuroimmune regulation, and inflammatory disorders [38, 39]. Furthermore, SIGLEC9 has been shown to influence neutrophil survival and inflammatory responses in highly activated immune environments, highlighting its complex immunomodulatory functions [40, 41].

The present findings are consistent with growing evidence implicating immune dysregulation and chronic inflammation in the pathogenesis of MDD [13, 42, 43]. By integrating MR with eQTL, pQTL, and Bayesian colocalization analyses, the present study provides complementary genetic evidence supporting SIGLEC9 as a candidate druggable gene associated with MDD.

Several therapeutic agents have been reported to target pathways involving members of the SIGLEC family, particularly in oncology and immune‐mediated diseases rather than neuropsychiatric disorders [44, 45, 46]. Therefore, the current findings should be interpreted as providing genetic support for future investigation rather than direct evidence of therapeutic efficacy in MDD.

Overall, SIGLEC9 represents a biologically plausible candidate gene linking immune regulation and depression. Nevertheless, further experimental studies are required to clarify its mechanistic role in MDD and to determine whether modulation of SIGLEC9‐related pathways may offer therapeutic benefit in clinical settings.

### 4.3 Immune Function of PSMB9 in MDD

PSMB9 is a catalytic component of the immunoproteasome and plays important roles in protein degradation, antigen processing, and immune regulation. In the present study, PSMB9 showed significant associations with MDD in both eQTL and pQTL analyses across blood and brain tissues, suggesting a potential role in disease pathogenesis. Previous studies have implicated immune activation, inflammatory signaling, and dysregulated antigen presentation in depression and other neuropsychiatric disorders, providing biological plausibility for the observed association [47].

In addition to its role in immune regulation, PSMB9 has been reported as a key component of immune‐related pathways in several neuropsychiatric conditions, including post‐traumatic stress disorder (PTSD) [47]. These findings support the hypothesis that alterations in proteasome‐mediated immune processes may contribute to psychiatric disease susceptibility. The present results further extend this evidence by providing genetic support for an association between PSMB9 and MDD.

Several proteasome inhibitors, including carfilzomib, have been reported to modulate pathways involving PSMB9 and are primarily used in the treatment of hematological malignancies rather than neuropsychiatric disorders [48]. However, the potential therapeutic relevance of these agents in MDD remains unknown. Furthermore, proteasome inhibitors are associated with clinically important adverse effects, including immunosuppression, cardiovascular toxicity, and renal dysfunction, which may limit their translational potential in psychiatric disorders [49].

Compared with previous studies that mainly focused on inflammatory and immune mechanisms in MDD, the present study provides complementary genetic evidence supporting PSMB9 as a candidate druggable gene associated with MDD through integrated MR, eQTL, pQTL, and Bayesian colocalization analyses [42, 43]. Nevertheless, the present findings do not establish PSMB9 as a validated therapeutic target. Further mechanistic studies and experimental validation are required to clarify its biological role and therapeutic potential in depression.

### 4.4 Immune Function of BTN3A1 in MDD

BTN3A1 (Butyrophilin Subfamily 3 Member A1) is a transmembrane protein belonging to the immunoglobulin superfamily and is involved in the regulation of immune cell activation, inflammatory responses, and adaptive immunity. In the present study, genetically predicted BTN3A1 expression was significantly associated with MDD in both blood and brain datasets, suggesting a potential role in the immune‐related mechanisms underlying depression.

Previous studies have shown that immune dysregulation and chronic inflammation contribute to the pathophysiology of MDD. BTN3A1 has been implicated in T‐cell regulation and Type I interferon signaling, both of which are important components of immune homeostasis [50]. Furthermore, BTN3A1 is located within the major histocompatibility complex (MHC) region, a genomic region that has been associated with several neuropsychiatric disorders, including schizophrenia [51]. These observations provide biological plausibility for the association identified in the present study.

Recent studies have also suggested that BTN3A1 may contribute to the shared genetic architecture of depression and schizophrenia. For example, Tao et al. reported BTN3A1 as a potential comorbidity locus and identified significant associations through summary‐data‐based MR analyses [52]. The present findings are generally consistent with these observations and provide additional genetic evidence supporting the involvement of BTN3A1 in psychiatric disorders.

Several therapeutic agents targeting BTN family proteins have been investigated in oncology and immune‐related disorders, reflecting the important immunomodulatory functions of this protein family [53, 54]. However, the relevance of these agents to MDD remains unclear, and their efficacy and safety in psychiatric populations have not been established. Therefore, the present findings should be interpreted as providing genetic support for future investigation rather than direct evidence of therapeutic applicability.

Overall, BTN3A1 represents a biologically plausible candidate gene linking immune regulation and MDD. Nevertheless, further mechanistic studies and experimental validation are required to clarify its functional role and to determine whether modulation of BTN3A1‐related pathways may have therapeutic relevance in depression.

### 4.5 Potential Role of TAPBP in MDD

TAPBP is an important component of the MHC Class I antigen presentation pathway. It facilitates peptide loading and stabilization of MHC Class I molecules through interactions with transporter associated with antigen processing (TAP) proteins. In the present study, TAPBP showed significant associations with MDD in both blood and brain datasets, suggesting a potential role in immune‐related mechanisms underlying depression.

Increasing evidence indicates that immune dysregulation and chronic inflammation contribute to the pathogenesis of MDD. Given its central role in antigen processing and presentation, TAPBP may influence immune signaling pathways relevant to neuroinflammation and immune homeostasis. Previous studies have reported altered methylation and expression patterns of TAPBP in mood disorders and other neuropsychiatric conditions, supporting its potential involvement in psychiatric disease susceptibility [55, 56, 57].

Furthermore, TAPBP has been identified among shared molecular signatures in several neurological and psychiatric disorders. Hossain et al. reported TAPBP as a shared hub protein between Parkinson's disease and bipolar disorder, highlighting its potential role in immune‐related biological pathways [56]. In addition, Murphy et al. identified differential methylation of TAPBP in cortical brain regions from individuals with MDD who died by suicide, providing further evidence supporting its relevance to depressive disorders [57]. Compared with previous studies that mainly reported associations between TAPBP and immune‐related mechanisms in neuropsychiatric disorders, the present study provides complementary genetic evidence supporting TAPBP as a candidate druggable gene associated with MDD through integrated MR, eQTL, pQTL, and Bayesian colocalization analyses [55, 56, 57]. Nevertheless, the current findings do not establish a direct therapeutic role for TAPBP. Further experimental studies are required to clarify its biological functions and to determine whether modulation of TAPBP‐related pathways may have therapeutic relevance in depression.

### 4.6 Comparison With Previous Studies and Biological Implications

Overall, the identified genes converge on biological pathways related to immune regulation, inflammation, antigen presentation, oxidative stress, and cellular signaling. These findings are generally consistent with previous genetic, transcriptomic, and experimental studies that have highlighted immune dysregulation and neuroinflammation as important contributors to the pathogenesis of MDD. However, compared with previous studies that primarily reported disease‐associated pathways or differentially expressed genes, the present study integrates MR, eQTL, pQTL, colocalization, and phenome‐wide analyses to prioritize genetically supported candidate druggable genes. Notably, MAPK3, MST1, and SIGLEC9 demonstrated consistent associations across multiple datasets, while PSMB9, BTN3A1, and TAPBP further support the involvement of immune‐related mechanisms in depression. Although these findings strengthen the evidence linking immune pathways to MDD, further experimental validation and clinical investigation are required to establish their biological and therapeutic relevance.

### 4.7 Study Limitations and Future Directions

Several limitations should be considered when interpreting the findings of this study. First, the analyses were based on summary‐level GWAS, eQTL, and pQTL datasets, which limited the ability to perform detailed individual‐level analyses and may affect the precision of causal inference. Second, although multiple sensitivity analyses were conducted to minimize potential bias, residual pleiotropy, unmeasured confounding, and violations of MR assumptions cannot be completely excluded. Third, the identified associations were derived from blood and brain molecular datasets, and some genes may exhibit tissue‐specific regulatory effects. Therefore, the biological interpretation of these findings should be approached with caution. In addition, most publicly available datasets used in the present study were generated from populations of predominantly European ancestry, which may limit the generalizability of the findings to other ethnic groups. Another important limitation is that the present study relied primarily on genetic association analyses. Although MR and colocalization analyses provide evidence supporting potential causal relationships, these approaches do not directly demonstrate biological mechanisms, therapeutic efficacy, or clinical safety. As this study was designed as an exploratory genetic target‐prioritization analysis, candidate genes were selected using an integrative framework based on multiple complementary analyses. Future studies using independent validation datasets and functional experiments will further strengthen the robustness of the identified candidate genes. Furthermore, the current findings have not yet been validated through cellular experiments, animal studies, or clinical investigations. Therefore, the identified genes should be considered candidate druggable genes requiring further experimental and clinical validation. Future studies should integrate additional omics datasets, including metabolomics, epigenomics, and single‐cell transcriptomics, to obtain a more comprehensive understanding of the molecular mechanisms underlying MDD. Functional studies using cellular and animal models will be important to clarify the biological roles of MAPK3, MST1, SIGLEC9, PSMB9, BTN3A1, and TAPBP in depression‐related pathways. In addition, future research should evaluate whether modulation of these targets can provide therapeutic benefit while maintaining acceptable safety profiles. Such efforts may facilitate the development of more precise and biologically informed treatment strategies for MDD.

Differences observed between blood and brain datasets may reflect tissue‐specific gene regulation. Gene expression patterns and biological functions often vary across tissues, particularly for immune‐related genes. Therefore, opposite effect directions do not necessarily indicate contradictory findings but may reflect distinct biological roles in peripheral immune cells and the central nervous system. Future studies using cell‐type‐specific datasets may help clarify these tissue‐dependent effects.

## 5 Conclusion

This study integrated MR with eQTL and pQTL data to prioritize several candidate druggable genes associated with MDD. These genes are involved in biological processes related to neuroinflammation, immune regulation, and apoptosis, supporting the growing evidence that immune‐related mechanisms contribute to the pathogenesis of MDD. Among the identified genes, MAPK3, MST1, and SIGLEC9 showed consistent associations across multiple analyses and emerged as genetically supported candidate targets for future investigation.

However, the present findings are based primarily on genetic association analyses and do not directly demonstrate therapeutic efficacy or safety. Therefore, further functional studies, experimental validation, and clinical investigations are required to clarify the biological roles and therapeutic potential of these targets. Future research integrating multi‐omics approaches and mechanistic studies may help translate these findings into more precise and biologically informed treatment strategies for MDD.

## Funding

This study was supported by Research Project of Yangpu District Technical Committee and Health Commission in Shanghai (YPGWQ202505, YPM202315, YPQ202405).

## Conflicts of Interest

The authors declare no conflicts of interest.

## Supporting information


**Table S1:** Druggable genes from the Drug‐Gene Interaction Database (DGIdb).
**Table S2:** Druggable genes from the study by Finan et al.
**Table S3:** Union of druggable genes.
**Table S4:** After filtering, the instrumental variables of druggable genes used for this study in the blood eQTL dataset.
**Table S5:** After filtering, the instrumental variables of druggable genes used for this study in the brain eQTL dataset.
**Table S6:** After filtering, the instrumental variables of druggable genes used for this study in the blood pQTL dataset.
**Table S7:** After filtering, the instrumental variables of druggable genes used for this study in the brain pQTL dataset.
**Table S8:** The results of the MR analysis between blood eQTL and GWAS data.
**Table S9:** The results of the MR analysis between brain eQTL and GWAS data.
**Table S10:** The results of the MR analysis between blood pQTL and GWAS data.
**Table S11:** The results of the MR analysis between brain pQTL and GWAS data.
**Table S12:** The results of the coloc analysis between candidate gene/protein and GWAS data.
**Table S13:** The instrumental variables of six prior druggable genes used for Phe‐MR analysis.
**Table S14:** 753 UKBiobank phenotypes for Phe‐MR analysis.
**Table S15:** The potential side effects or additional indications of prior druggable genes(FDR value < 0.05).

## Data Availability

Data available on request from the authors.
