# Supplementary material for: Multi‐Omics Mendelian Randomization Prioritizes Candidate Druggable Genes for Major Depressive Disorder
Source: Genes Brain Behav. 2026 Nov 9;25(6):e70079. doi: 10.1111/gbb.70079 (PMC13748815; doi:10.1111/gbb.70079)
Supplement: Supplementary file 1 [file GBB-25-e70079-s001.zip › gbb70079-sup-0001-TableS1-S15.docx]

***Supplementary material***

Additional supporting information may be found in the Supporting Information file of the article.

Table S1	Druggable genes from the Drug-Gene Interaction Database (DGIdb).

Table S2	Druggable genes from the study by Finan et al.

Table S3	Union of druggable genes

Table S4	After filtering, the instrumental variables of druggable genes used for this study in the blood eQTL dataset.

Table S5	After filtering, the instrumental variables of druggable genes used for this study in the brain eQTL dataset.

Table S6	After filtering, the instrumental variables of druggable genes used for this study in the blood pQTL dataset.

Table S7	After filtering, the instrumental variables of druggable genes used for this study in the brain pQTL dataset.

Table S8	The results of the MR analysis between blood eQTL and GWAS data.

Table S9	The results of the MR analysis between brain eQTL and GWAS data.

Table S10	The results of the MR analysis between blood pQTL and GWAS data.

Table S11	The results of the MR analysis between brain pQTL and GWAS data.

Table S12	The results of the coloc analysis between candidate gene/protein and GWAS data.

Table S13	The instrumental variables of six prior druggable genes used for Phe-MR analysis.

Table S14	753 UKBiobank phenotypes for Phe-MR analysis.

Table S15	The potential side effects or additional indications of prior druggable genes(FDR value < 0.05).
